# Procrastinating Behavior in Computer-Based Learning Environments to Predict Performance: A Case Study in Moodle

**DOI:** 10.3389/fpsyg.2017.01403

**Published:** 2017-08-24

**Authors:** Rebeca Cerezo, María Esteban, Miguel Sánchez-Santillán, José C. Núñez

**Affiliations:** Facultad de Psicología, Universidad de Oviedo Oviedo, Spain

**Keywords:** procrastination, CBLEs, learning failure, Educational Data Mining

## Abstract

**Introduction:** Research about student performance has traditionally considered academic procrastination as a behavior that has negative effects on academic achievement. Although there is much evidence for this in class-based environments, there is a lack of research on *Computer-Based Learning Environments (CBLEs)*. Therefore, the purpose of this study is to evaluate student behavior in a blended learning program and specifically procrastination behavior in relation to performance through Data Mining techniques.

**Materials and Methods:** A sample of 140 undergraduate students participated in a blended learning experience implemented in a Moodle (Modular Object Oriented Developmental Learning Environment) Management System. Relevant interaction variables were selected for the study, taking into account student achievement and analyzing data by means of association rules, a mining technique. The association rules were arrived at and filtered through two selection criteria: 1, rules must have an accuracy over 0.8 and 2, they must be present in both sub-samples.

**Results:** The findings of our study highlight the influence of time management in online learning environments, particularly on academic achievement, as there is an association between procrastination variables and student performance.

**Conclusion:** Negative impact of procrastination in learning outcomes has been observed again but in virtual learning environments where practical implications, prevention of, and intervention in, are different from class-based learning. These aspects are discussed to help resolve student difficulties at various ages.

## Introduction

Research on self-regulated learning (SRL) behavior covers a wide area of knowledge. It has shown us that learners of all ages have difficulties deploying key cognitive and metacognitive self-regulatory skills during learning in open-ended learning environments (Azevedo, 2015) like many *Computer-Based Learning Environments* (CBLEs). CBLEs have brought new opportunities to current education (European Commission, 2014) but also bring many challenges for the student. Deciding what, when, how, and for how long to learn, in short, self-regulation, gains importance in this context (Lajoie and Azevedo, 2006; Jacobson, 2008; Winters et al., 2008; Azevedo et al., 2009; Michinov et al., 2011; Klingsieck et al., 2012; You, 2015; Sánchez-Santillán et al., 2016). In this study, we specifically explore a small but determinant part of self-regulation, procrastination, trying to discover its relationship with student failure in CBLEs. Although there is little work on this specific topic, we provide an overview of the key concepts and related research and then try to shed some light on our research questions through the so-called Data Mining methodology Association Rules. Lastly, we propose several ways to use our findings to improve student learning and avoid academic failure.

### Self-Regulated Learning, Time Management, and Procrastination in Academic Contexts

SRL involves deploying metacognitive, motivational, and behavioral processes in a systematic way, being able to adapt strategies *to different contexts*, in order to achieve the stated learning goals (Zimmerman, 1990); in this particular case, we would say *to different learning environments* like the increasingly common open-ended CBLEs. Self-regulated students face the learning process with confidence, draw up a plan to guide the study, monitor processes, adapt them to suit changing environments, and know when they have achieved their goals (Zimmerman, 1990). Bearing in mind the complexity of the SRL construct, in this paper, we focus on one of the dimensions of SRL, time management.

Zimmerman and Risemberg observed as early as 1997 that within the personal qualities that differentiate students who succeed from those who do not, there are six underlying self-regulatory processes; time use, goal setting, self-monitoring, self-reactions, self-efficacy, and motivation (Zimmerman and Risemberg, 1997). From these processes, time management, motivation, and perceived self-efficacy play the most important role (Zimmerman, 1998). Along the same lines, several studies highlight the impact of time management on fear of failure and motivation (Visser et al., 2015), anxiety and stress (Hen and Goroshit, 2014; Häfner et al., 2015) and academic achievement (Balkıs, 2011; You, 2015). Therefore, it seems as though time management plays a notable role in educational outcomes from school to higher education and is highly interconnected with many others variables that somehow determine learning (Zimmerman and Risemberg, 1997; Zimmerman, 1998; Liu et al., 2002; Lee, 2005; Stoeger and Ziegler, 2008; Odacı and Kalkan, 2010; Rabin et al., 2011; Kirk et al., 2013; Visser et al., 2015). Thus, although it is possible for students to acquire time management and other self-regulation skills through proper intervention, most continue with this handicap throughout their education, and it is often pointed out as a skill lack that affects students from primary to tertiary education (Reid and Moore, 2008), and in authentic and online learning environments (Lewis et al., 2014; Shukla et al., 2014).

Numerous studies report on the importance of time management and learning, not only in terms of quantity but also the quality of time the students spend learning (Balkıs, 2011). Many of these studies focus on academic procrastination, understood as “the tendency to postpone an activity under one’s control to the last possible minute or even not to perform it at all” (Gafni and Geri, 2010, pp. 115) and extensively researched for decades. One of the seminal empirical papers on student procrastination was published by Beswick et al. (1988). In the last few decades, several different approaching to procrastination arose. While some authors see functional forms of procrastination (e.g., Chu and Choi, 2005), others take the view that procrastination has no functional aspects (e.g., Corkin et al., 2011). In a review of procrastination construct typology attempts, Gueorguieva (2011) maintained that there are different theorists using different labels when referring to similar types of procrastination but what it is already well known is that failures in self-regulation are the core of academic procrastination and that this phenomenon poses a serious threat to students’ academic achievement and subjective well-being (Steel and Klingsieck, 2016).

In addition, procrastination is one of the most extended lapses in time management and is a common student behavior in every educational stage (Terry, 2002; Rabin et al., 2011; Yang, 2012; Romero, 2013; Katz et al., 2014; Karatas, 2015). For instance, Sánchez (2010) found the presence of these behaviors in 80% of university students and found that it was chronic in 20% of them. Hence, procrastinating behavior—even though it is a common practice in modern western societies—is in need of further research (Levy and Ramim, 2012).

As mentioned previously, the negative effect of procrastination on learning and performance has been observed in authentic educational settings (class-based learning) but there is a lack of research within CBLEs, which is aggravated because, as has been previously observed, procrastination has even greater influence in distance learning settings (Tuckman, 2005). This kind of misbehavior also seems to be related to the higher student dropout rates in online than conventional learning environments. In order to explain or predict dropout in online courses, different conceptual models were suggested (Lee et al., 2013; Cochran et al., 2014). These approaches found several predictors of dropout associated with the difficulty of employing responsible self-generated academic behavior in these environments (Azevedo et al., 2009), leading to the conclusion that a student who displays self-regulation skills is more likely to succeed in CBLE one who does not (Winters et al., 2008).

Returning to previous results specifically related to online learning, Michinov et al. (2011) found that high procrastinators are less successful online learners than low procrastinators. Recently, You (2015) applied multiple regression techniques over LMS data from 569 college students obtaining results that emphasize time management as a predictor of course achievement. However, both studies concluded that although their work sheds some light on the relationship between procrastination and performance, further research is necessary to expand the understanding of procrastination in online learning environments.

Related results were also produced by Goda et al. (2015) in a longitudinal study with the goal of observing university students’ learning behavior in an e-learning environment. The authors found seven behavioral profiles (procrastination, learning habit, random, diminished drive, early bird, chevron, and catch-up) and their relation to learning outcomes, highlighting the better performance of students with a learning habit profile, in contrast to those with a procrastinating profile. Meanwhile, Broadbent and Poon (2015) carried out a useful literature review of SRL strategies and academic achievement in online learning environments emphasizing the association of optimal time management and academic success in almost all research reviewed. Moreover, several studies confirm the association of procrastination and other academic misconduct, with the most frequent being the use of fraudulent excuses (Patrzek et al., 2015; Sureda-Negre et al., 2015).

Although some research findings highlight the negative effect of procrastination, others have pointed out an active procrastination profile corresponding to students who decide to postpone tasks in order to produce a better performance (Choi and Moran, 2009; Kim and Seo, 2013). This kind of finding, along with the observed consequences of procrastinating behavior in general learning, make it even more necessary to contextualize the study of this specific phenomena in open-ended CBLEs.

### EDM and Association Rules

CBLEs present significant differences in relation to traditional learning settings that should be taken into account but that can also offer an advantageous environment to observe and overcome their aforementioned challenges; In general, CBLEs are ready to collect large amounts of data from the user–machine interaction. In particular, LMSs collect data from students that, properly analyzed, can provide teachers and researchers with the necessary information to support and constantly improve the learning process (García et al., 2009; Paule-Ruiz et al., 2015). One of the most used is Modular Object Oriented Developmental Learning Environment (Moodle), a free LMS enabling the creation of powerful, flexible, and engaging online courses and experiences (Rice, 2006). Unfortunately, these platforms do not provide specific tools to allow educators to thoroughly track and assess all students’ learning process but one of the most suitable promising and innovative techniques for handling these data is based on Educational Data Mining (EDM).

EDM is an interdisciplinary research field, developing methods for exploring the unique data that come from computer educational environments (Romero and Ventura, 2013). Different EDM procedures have been used to get a better understanding of the underlying educational processes, to generate recommendations for students, to provide feedback to either students, teachers, or/and researchers, to early detect learning difficulties, to help students with specific learning disabilities, to avoid academic failure, etc.; in short, to help address the difficulties that students of different ages have when learning in highly cognitively and metacognitively demanding learning environments, like open-ended CBLEs (García et al., 2009; Azevedo et al., 2012). Previous research has shown how web usage mining can be applied in Moodle in order to predict the marks that students will obtain in a course (Romero et al., 2013) and even specific Moodle mining tools have been developed for the use of not only experts in data mining but also of newcomers like instructors and courseware authors (Romero et al., 2008).

One of those procedures is the so-called *association rules*, one of the most commonly used and best known Data Mining techniques (Romero et al., 2010a) in very different research disciplines such as medicine (Antonie et al., 2001), earth sciences (Tan et al., 2001), banking (Aburrous et al., 2010), telecommunications (Wei and Chiu, 2002), and the stock-market (Hajizadeh et al., 2010), and also in the educational field. This methodology has been extensively used to identify e-learning indicators and their influence on student performance (Paule-Ruiz et al., 2015), describe learning behavioral profiles (Goda et al., 2015), point out variables that influence instruction (Romero et al., 2010a), to improve a collaborative learning experience (Mora et al., 2014), to test 3D virtual reality environments (Cherenkova et al., 1996), to understand the role of social networks in learning (Paredes and Chung, 2012), and as the basis of adaptive learning systems (Murugananthan and ShivaKumar, 2016). Based on this body of previous research, and as Han already concluded in 2001, it seems as though this methodology could produce enough knowledge to discover patterns from a huge amount of data which would be a useful base for a decision-making process (Han and Kamber, 2001).

In this paper, we intend to apply such analysis techniques to data gathered from a course implemented in an open-ended learning environment managed by a Moodle system in order to discover time management parameters which will, hopefully, be stable over time and samples that could be used as predictors of the learning process and its result.

### Research Questions

Considering the limited previous research findings in this particular area, we arrive at the starting research question, does procrastination behavior have any predictive value for the student’s performance in LMSs in a distance learning experience? Supported by previously reviewed literature in face-to-face learning environments we hypothesize that procrastinating behavior will have that assumed predictive value. Secondly, and if so, how is procrastinating behavior related to student performance in the LMS? Paule-Ruiz et al. (2015) and You (2015, 2016) found results in this direction but with a very different methodology based on correlation, not necessarily causation. Moreover, although these research findings found a negative effect of procrastination, others have suggested the opposite (Choi and Moran, 2009; Kim and Seo, 2013), leading us to hypothesize with less confidence than the first hypothesis but predicting a negative relationship between procrastination and achievement, and therefore making further research necessary.

Finally, regarding the hypothetical Association Rules’ predictions made for a given sample, are they stable enough to apply to another sample in following academic years? In other words, we expect to extrapolate from the hypothetical predictive values for procrastinating behaviors and use them to predict different student’s performance in LMSs?

## Materials and Methods

### Participants

To test our research questions, we applied EDM techniques to log file data from a Moodle 2.0 course, 140 undergraduate psychology students from a state university in Northern Spain took part in this research through a Blended-learning course. The sample was formed mainly by women (83%), as the population of psychology students is highly feminized. Their ages at the moment of the study were ranged between 19 and 21 years (mean age = 20.23; SD = 1.01).

This research focuses its attention on the study of *variables related to effort and time spent working*—also needed in order to ensure that students perform the minimum requested tasks—and *variables related to procrastinating behavior* as focal parameters of student interaction with the LMS used in the current work.

### Procedure

We analyzed the interaction of two groups of undergraduate psychology students with an LMS over two consecutive academic years (N_1_ = 67; N_2_ = 73). The course is *eTraining for Autonomous Learning—eTRAL program* (Cerezo et al., 2010; Núñez et al., 2011) implemented in a university in the North of Spain. eTRAL is a program about SRL and study strategies that take part of the course curriculum but completed entirely outside of teaching hours, and organized into 11 weeks/blocks (blended-learning). Every Monday a new block is accessible to the students, allowing them a 2-week period to complete it. In order to do so, the students ought to carry out three compulsory tasks per block, in any order: First, check a *theoretical* content about learning strategies; second, complete a *practical task* related to the theoretical content; third, contribute with a post in the subsequent *forum*. These tasks merged with the three levels of knowledge to reach an optimal learning (Biggs, 2005): declarative knowledge level: theoretical contents, description, information, and how-to put in practice the strategy or strategies of the week; procedural knowledge level: practical tasks where the students have to put the declarative knowledge into practice; conditional knowledge level: discussion forums where the students have to discuss about how they have or would use the strategy or strategies of the week in different contexts. The role of the instructor was to manage the Moodle course interfering as less as possible in the learning process; just setting the technical details required for running the contents, notifying by mail every time that there were a new unit available—even though it follows a feasible periodicity—moderating the forum if necessary, and answering students questions off-line about technical o theoretical issues.

Due to eTRAL being part of the course content, students were required to complete 80% of the 11 blocks in order to gain an extra point in the final mark of the course. Further information about this program can be found in Cerezo et al. (2015).

### Extraction of the Variables

During the implementation of the course, the interaction of the students with the LMS is recorded in the Moodle database logs (Cole and Foster, 2007). The Moodle system tracks student interaction based on actions collected from every student and their metadata, for example; the date, kind of action, and name of the resource which has been worked on. Moodle stores a total of 76 actions, but we selected only eleven raw log actions (see column *Moodle Actions* on **Table 1**), paying particular attention to previously contrasted significant variables of students’ interaction with LMSs (Hung and Zhang, 2008; Lust et al., 2012, 2013; Macfadyen and Dawson, 2012; Murray et al., 2012; Kim et al., 2014; Cerezo et al., 2016), and particularly representative of the students’ performance in this Moodle course (Cerezo et al., 2016), which allows us to recalculate nine representative variables for our study. A few variables were extracted directly from Moodle records; however, it is sometimes advisable to formulate queries to obtain aggregated results (Talavera and Gaudioso, 2004) so other variables were calculated based on those records with a simple operation (e.g., as seen in **Table 1**, the variable *Days Post* is calculated by subtracting the date that the student *Posts* their opinion in the forum from the date that is officially possible to *View* and *Post* in the forum).

**Table 1:** Name of variables considered in the study with their description and extraction method.

Variable	Description	Moodle actions	Extraction method
**Variables related to effort and time spent working**			
Time Theory	Minutes spent on theoretical contents	*Resource view*	Sum of the periods between *resource view* and the next different action
Time Task	Minutes spent on practical tasks	*Quiz view, quiz attempt, quiz continue attempt, quiz close attempt*	Sum of the periods between *quiz view/quiz attempt/quiz continue attempt/quiz close attempt* and the next different action
Time Forum	Minutes spent in forums	*Forum view, forum view discussion, forum add reply, forum add discussion, forum add post, update post*	Sum of the periods between *forum view/forum view discussion/forum add reply/ forum add discussion/forum add post/update post* and the next different action
Relevant Actions	Total of significant actions in the LMS	All actions related to Time Theory, Time Task, and Time Forum	Actions like log in, log out, profile updating, check calendar, refresh content, etc. are dismissed
**Variables related to procrastination behavior**			
Days Theory	The days that go by from when a block is available until the student checks the theoretical contents for the first time	Date of the first *resource view* after the block became available	Date of *resource view* after the theoretical contents became available
Days Task	The days that go by from when a block is available until the student checks the practical task for the first time	Date of the first *quiz view* after the block became accessible	Date of *quiz view* since the task became available
Days “hand-in”	The days that go by from when a block is unlocked until the student finishes the task	Date of the first *quiz close attempt* after the block was made available	Date of *quiz close attempt* after the task became available
Days Forum	The days that go by from when a block is unlocked until the student checks the forum for the first time	Date of the first *forum view discussion* after the block was made available	Date of *forum view discussion* after the task became available
Days Post	The days that go by after a block is unlocked until the student writes in the forum for the first time	Date of the first *forum add reply* after the block was made available	Date of *forum add reply* after the task became available

The variables were extracted and organized in two different groups taking into account what they represent at a higher granularity level: *variables related to effort and time spent working* and *variables related to procrastination:*

• *Variables related to effort and time spent working.* One of the most challenging issues was giving meaning to the learning context from the raw data. Aiming to achieve this, we have classified the variables *Time Theory, Time Task, Time Forum*, and *Relevant Actions* in this group as indirect indexes of student effort.
• *Variables related to procrastination.* On the one hand, the number of days in a 2-week period that the students wait to check each assignment, the task (*Days Task*), the forum subject (*Days Forum*) and the theoretical content (*Days Theory*). On the other, the number of days that they take to hand in the task (*Days hand-in*) and post their opinion (*Days Post*). The rationale was that we wanted to approach procrastination by observing the students’ behavioral patterns before the homework deadline and not solely considering late or absent submissions.

In summary, nine student interaction variables from the LMS (**Table 1**) were extracted along with student achievement in this course, used as the tenth variable.

### Data Analysis

Class Association Rules (CAR) were applied to the described data. The CAR are a variety of association rules which allow the identification of confluent relationships between a combination of variables and a class variable pre-defined by the researcher. Thus, the association rules are defined by the conditional relation of one of the characteristics to be analyzed (precedent variables) and the previously defined class, which would be the consequent (IF the precedent variable takes place, THEN it is revealed in the categorization of the subject in a given class) (Romero et al., 2010b). Rule interest is based on support and confidence measures (Hastie et al., 2001). Support denotes how frequently the precedent appears in the dataset (Hahsler et al., 2005). Confidence denotes how often the rule appears in the dataset (Hipp et al., 2000).

In this process, we have used the Predictive Apriori algorithm. This algorithm searches with an increasing support threshold for the best n rules concerning a support-based corrected confidence value (Scheffer, 2001). Predictive Apriori considers both the confidence and support in ranking the rules. A Bayesian framework is used to calculate the predictive accuracy out of the support and confidence of a rule (Nahar et al., 2013). Predictive accuracy values are between 0 and 1. Predictive Apriori has been chosen because, in general, it performs better than the Apriori algorithm (García et al., 2011). In order to produce the rules, Weka (Hall et al., 2009), the software used for analysis, needs to receive discrete variables. Discretization is a process that transforms numeric variables into categorical variables (Hussain et al., 1999). Equal-width is a method that discretizes the domain of a variable into equal-width intervals (Chmielewski and Grzymala-Busse, 1996). In the present study, we have selected equal-width method to discretize antecedent variables as seen in previous work (García et al., 2011; Paule-Ruiz et al., 2015). Also, performance was selected as class variable (consequent) and was reasonably discretized based on Spanish typical grading system of students’ performance: from 0 to 4.9 points, we assigned the value “Low” (as it means that the student failed the course exam), from 5 to 6.9 points as “Medium” value, and from 7 to 10 points as “High” value (see **Table 2**). These values were extracted from the performance of every student. In this work, it is considered to be an index of general achievement because it is not only the grade for the assignments completed during the LMS e-course but also the sum of the grade with an objective final exam of the subject.

**Table 2:** Discretization method and discretized values for each variable.

Variable	Discretization method	Discretized values
Time Theory	Equal-width	Low, Medium, High
Time Task	Equal-width	Low, Medium, High
Time Forum	Equal-width	Low, Medium, High
Relevant Actions	Equal-width	Low, Medium, High
Days Theory	Equal-width	Early, Normal, Late
Days Task	Equal-width	Early, Normal, Late
Days “hand-in”	Equal-width	Early, Normal, Late
Days Forum	Equal-width	Early, Normal, Late
Days Post	Equal-width	Early, Normal, Late
Performance	Manual-Method	Low, Medium, High

## Results

In many cases, association rules algorithms generate a high number of association rules and it is nearly impossible for teachers to comprehend or validate such a quantity of rules (Kotsiantis and Kanellopoulos, 2006). As the objective was to predict student performance in upcoming years, and we had samples from two courses, we only selected the rules that were repeated in both years. This method allows us to validate the rules’ generalizability in order to apply the results to new students in similar contexts, as pointed by Winne and Baker (2013). As result of this procedure, we achieved rules describing behaviors that were consistent throughout the samples and academic years.

Application of the Predictive Apriori algorithm supplied 49 rules during the first academic year and 62 rules during the second academic year with an accuracy greater than 0.94, producing 111 rules in total. The number of times that each variable appears in the rules found, as well as the ratio between the previous and the number of rules found, is shown in **Table 3**.

**Table 3:** Variables’ distribution in the rules obtained for each academic year and their global presence.

Variable	First academic year	Second academic year	Both academic years
Antecedent variables			
Time Theory	33 (67.34%)	37 (59.67%)	70 (63.06%)
Time Task	25 (51.02%)	24 (38.70%)	49 (44.14%)
Time Forum	0	19 (30.64%)	19 (17.12%)
Relevant Actions	17 (34.69%)	18 (29.03%)	35 (31.53%)
Days Theory	15 (30.61%)	13 (20.96%)	28 (25.25%)
Days Task	16 (32.65%)	20 (32.25%)	36 (32.43%)
Days “hand-in”	15 (30.61%)	11 (17.74%)	36 (32.43%
Days Forum	15 (30.61%)	10 (16.12%)	25 (25.25%)
Days Post	19 (38.77%)	26 (41.93%)	45 (40.54%)
Consequent/class variable values			
Performance—High	12 (24.48%)	12 (19.35%)	24 (21.62%)
Performance—Medium	20 (40.81%)	19 (30.64%)	39 (35.13%)
Performance—Low	17 (34.69%)	31 (50%)	48 (43.24%)

*Natural numbers indicate the amount of rules containing each variable, and the percentage corresponds to its presence in the total amount of rules.*

Next, we merged the 111 rules obtained into one file and with a simple algorithm, we selected those ones that were repeated in both academic years. We considered a rule as repeated if it had the same precedent (same variables with same values), and it had the consequent class value (*Performance: Low, Medium, and High*). If a rule was repeated in both years, with the same precedent and consequent but there was another rule with the same precedent and different consequent value, this rule was automatically discarded by the algorithm. The algorithm found three association rules which are repeated in both academic years:

• *Days Theory = NORMAL and Days Task = LATE → Performance = LOW* (accuracy = 0.972). Rule 1 states that if the access to theoretical content is done in an average time and access to task is carried out late, then, performance is low.
• *Time Theory = LOW and Days Theory = LATE and Days Forum = NORMAL → Performance = LOW* (accuracy = 0.943). Rule 2 shows that if the average time devoted to theoretical content is low, access to the theoretical content is late and access to the forum is in an average time, then performance is low.
• *Time Task = MEDIUM and Days Theory = EARLY and Days Task = NORMAL → Performance = HIGH* (accuracy = 0.943). Rule 3 reflects how if the average time devoted to task fulfillment is medium, access to the theoretical content is early and access to task is in an average time, then performance is high.

## Discussion

This work focuses on procrastination, one of the most common problems at every educational level and is an extension of the similarly prevalent and pernicious phenomena in daily life (Steel, 2007). This failure of time management is more frequent when the learning process is not class-based (distance or computer-based learning), as the student has to take an active role, where self-regulation becomes determinant (Yaakub, 2000; Azar et al., 2009; Klingsieck et al., 2012). In this study, we have observed how procrastination behaviors can lead to poor academic results while learning in an LMS, something that has been previously and thoroughly noted in authentic academic contexts (Kim and Seo, 2015) but not as extensively in online learning environments (Michinov et al., 2011; You, 2015).

We tracked and analyzed student behavior in an LMS, specifically procrastination behaviors in relation to performance through Data Mining techniques. Relevant interaction variables were selected for the study, also taking into account student achievement and analyzing data by means of extracting and filtering association rules. The association rules obtained show the importance of academic procrastination when learning in distance CBLEs. At first sight, it can be seen that two out of the three variables making up the antecedents are related to procrastination behavior in most of the Rules. Moreover, in general terms, evidence of procrastination in the antecedents leads to poor performance, and signs of successful time management end up with satisfactory achievement. The global presence of the variables in the 111 Rules is also revealing. Looking again at **Table 3** it can be seen that three out of the five most commonly present variables belong to actions in the LMS related to procrastination behavior.

In particular, if we analyze Rule 1, we can see how when a student accesses the theoretical resource in an average time but delays dealing with the corresponding task, performance is lower. Looking at Rule 2, it shows that students that access the theoretical resource late, and devote a little time to it, but check the forum topic in an average time-frame, perform worse. It makes sense that when a student starts working late on a topic, they have less time available to take advantage of, and consequently achieve lower marks. Interpreting these results in terms of procrastination, they are more pessimistic but agree with those found by Paule-Ruiz et al. (2015), who found that when students start assignments late, they perform poorly, Michinov et al. (2011) that found that procrastination and performance in online learning environments was mediated by the level of the learners’ participation in discussion forums, and You (2015) found that the extent of achievement predictability of academic procrastination in LMSs cumulatively increased at different time points of the course. Therefore, this rule could be indicating that even students who start studying within an average time-frame are at risk of later procrastination behaviors and subsequent consequences in terms of performance. This interpretation could be very valuable considering that students who postpone and cram assignments at the last minute showed poorer long-term retention and achievement (Tuckman, 2005; Asarta and Schmidt, 2013). According to Bannert et al. (2014), there are differences in the temporal pattern of students’ spontaneous learning steps when learning in hypermedia environments, also in how their regulatory activities unfold over time. Therefore, early detection of low self-regulated learners is necessary to provide them with support at the right time. A key application of these results concerns personalization in e-learning environments, such as the suitability of different types and times of prompts for different students’ learning models (Lehmann et al., 2014) and building Recommender Systems based on e-Learner groups (Kardan et al., 2012). By combining the knowledge from this with previous work about learning in LMSs, this study could contribute to a valid student learning model for adaptive learning systems (Brusilovsky, 2001; Cerezo et al., 2016).

Rule 3 shows how a more organized learning process, around the average, can lead to academic success. When the student accesses the first theoretical resource early, spends a medium amount of time on assignments and accesses the assigned task within an average time-frame, they achieve high performance. It is remarkable that this is the only Rule with an early value in procrastination values and the only one with a satisfactory performance in the consequent. These findings agree with the conclusions of the meta-analysis carried out by Kim and Seo (2015), which found procrastination variables to be negatively correlated to students’ performance, but we dare say that the results of this study are more valuable for intervention in low achievers’ learning issues.

At this point, the found association rules lead us to clarify our two first research questions, supporting the idea that procrastination variables can be used to predict student performance (first research question) and that the values of procrastination variables are inversely related to student performance (second research question).

With respect to the third research question, about the association rules’ predictive potential, it seems as though that is solved by the methodology used itself. Although 111 rules merged from both samples, in two different academic years, the Rules discussed are the only ones present in both academic years, with the same precedent about time management variables and the same consequent in terms of performance. It seems that these indicators are steady over time (different course) and individuals (different samples) and so could potentially be used as predictors for the same course in following academic years benefiting students with knowledge obtained from previous cohorts. Similar practical implications were found by Sekhavatian and Mahdavi (2011), Mosharraf and Taghiyareh (2012), and Murugananthan and ShivaKumar (2016), who used this kind of predictors as a guide for their learning recommender system in subsequent years.

Considering that variables related to procrastinating behavior are present in every Rule and are two out of three variables that define the antecedent, it could be considered that the procrastinating behaviors in the precedent could have a predictive value for early detection of student performance in LMSs. In this sense, it seems as though EDM, and in particular the methodology used in the current study, will be able to contribute beyond strictly predicting student performance, as a guide to improve learning process efficacy, as claimed by Mosharraf and Taghiyareh (2012). Most procrastination studies, even in CBLEs, have used self-reported questionnaires to measure the behavioral tendencies of this phenomena, or statistical techniques such as correlations or multiple regression (You, 2015).

Although these instruments have been validated and the procedures used in many studies, they are intrusive for the students and limited to capturing the variables of interest during a course. In this sense, self-report measures could be not enough to measure a construct with a processual nature, apart from how the questions shape the answers, and other well-known limitations (Schwarz, 1999). Likewise, the current study methodology is one of the values of this research, not for adopting an EDM approaching able to capture the learning process but for applying CAR technique to the study of the procrastination in LMSs. LMSs collect data from students that, when properly analyzed, can provide the different educational agents with the necessary information to support and constantly improve the learning process (Paule-Ruiz et al., 2015). One of the most promising conclusions from this work would have been harder to be learned without using Data Mining techniques. The variables that most of the studies use to research about procrastination and could be expected to shed more light on the research questions were not that relevant in our study (last minute submissions, late submissions, failure completing assignments, etc.) (Michinov et al., 2011; You, 2015, 2016). In other words, approaching the procrastination phenomena as a result, finalization actions like hand-in the homework on time, or not, seems to be a feasible index of this behavior. However, none of those finalization actions defined the repeated Association Rules in subsequent samples in our study (Days hand-in and Days Post). In contrast to, variables belonging to the procrastination process, always previous to fail with a task deadline, were present, denoting the importance of approaching to learning as a process, not as a result. These particular results have an essential application to Adaptive Hypermedia Systems and Adaptive Educational Systems (De Bra and Calvi, 1998; Brusilovsky, 2001).

To sum up, these results seem to confirm the association of time management and academic achievement in the LMS, particularly for those behaviors denoting procrastination. Those students that perform their academic work early or with average timing, and devote a sufficient amount of time to it, demonstrate satisfactory performance, whereas those students that do not manage timing well (in terms of checking and devoting time to tasks and study) are unable to match the standards and perform worse. Similar results have been obtained by many researchers, regardless of country, educational level, or educational setting (Tuckman, 2005; Stoeger and Ziegler, 2008; Tan et al., 2008; Liu et al., 2009; Rakes and Dunn, 2010; Balkıs, 2011; Michinov et al., 2011; Broadbent and Poon, 2015; Goda et al., 2015; Paule-Ruiz et al., 2015). Therefore, in combination with the aforementioned Adaptive and Educational Hypermedia Systems technology, and knowing that these learning environments can be more challenging for students both with and without learning difficulties (Rodríguez-Málaga et al., 2017), and understanding that procrastination is a failure in academic self-regulation (Clariana et al., 2011), empowerment of SRL in open-ended CBLEs is the key. There is already well-studied software which is able to perform assessment and training in a wide spectrum of SRL [e.g., about epistemic beliefs (Trevors et al., 2016), reading patterns (Bondareva et al., 2013), scaffolding (Azevedo et al., 2010), learning strategies (Trevors et al., 2014), self and social-regulation (Azevedo, 2014), emotions (Azevedo et al., 2013), motivation (Duffy and Azevedo, 2015), and engagement (Azevedo, 2015)], among other things, but at this point it is necessary to work together with computer science to develop reliable prediction models and efficient preventive tools.

Although these results shed light on the phenomenon being studied, several limitations should be noted. With respect to the results of Choi and Moran (2009) and Kim et al. (2014), still further research is needed to determine which procrastination variables could be linked to the different active and passive procrastination profiles found. Regarding methodology, the online learning experience is a core variable to be controlled in future research. However, the context of the present work was a traditional university, and CBLEs have become more conventional, so students are assumed to have similar levels of online learning experience. In addition, LMSs are only one component of the learning ecosystem (García-Peñalvo and Seoane Pardo, 2015). This raises awareness about the future work on data collection moving toward Personal Learning Environments (PLEs) or Massive Open Online Courses (MOOCs), and checking the results in diverse types of learning platforms. Moreover, it would be more appropriate to compare two student groups who studied the course in the same academic year synchronically than over two consecutive years, however, using different sets of data helps to validate the rules’ generalizability in order to apply the results to new students in similar contexts. In this line, we have generated valid and consistent rules selecting the repeatedly discovered ones in both samples; this is only the first and previous step to apply those rules to predict the performance in other student groups and check its accuracy, a very close prospect of the present work. Finally, it is well known that novice students report less sophisticated study strategies to address new domains of information (Alexander et al., 2004) so the results of this study could have been different if it had been in freshman students.

To conclude, this study sheds some light on the relationship between procrastination and performance in open-ended learning environments and provides interesting possibilities for improving online learning together with fruitful material for future research.

## Ethics Statement

The research design was developed in accordance with the Declaration of Helsinki and the Spanish Law of Personal Data Protection (15/1999) principles. The data examined in this study was covered by the permission that every student of our university gives on the enrolment moment every year, a procedure supported by *The Ethics Committee for Research at Universidad de Oviedo*. In addition, participation in our study was voluntary and experimenters informed students about data usage.

## Author Contributions

RC contributed to the design of the study and data interpretation. She also took part in the writing process of the manuscript, research questions setting and interpretations and discussion of the results. JN coordinated the research and gave the final approval of the manuscript to be submitted. ME drafted the work and critically reviewed the theoretical background and conclusions. MS-S was involved in the design of the work as well as the acquisition and data analysis.

## Conflict of Interest Statement

The authors declare that the research was conducted in the absence of any commercial or financial relationships that could be construed as a potential conflict of interest.

## References

[B1] Aburrous M. Hossain M. A. Dahal K. Thabtah F. (2010). Intelligent phishing detection system for e-banking using fuzzy data mining. *Expert Syst. Appl.* 37 7913–7921. 10.1016/j.eswa.2010.04.044

[B2] Alexander P. A. Sperl C. T. Buehl M. M. Fives H. Chiu S. (2004). Modeling domain learning: profiles from the field of special education. *J. Educ. Psychol.* 96 545–557. 10.1037/0022-0663.96.3.545

[B3] Antonie M. L. Zaiane O. R. Coman A. (2001). “Application of data mining techniques for medical image classification,” in *Proceedings of the Second International Conference on Multimedia Data Mining* eds Zaïane R. Simoff J. (Berlin: Springer-Verlag) 94–101.

[B4] Asarta C. J. Schmidt J. R. (2013). Access patterns of online materials in a blended course. *Decis. Sci. J. Innov. Educ.* 11 107–123. 10.1111/j.1540-4609.2012.00366.x

[B5] Azar I. Hinojo F. J. Cáceres M. P. (2009). Percepciones del alumnado sobre el blended learning en la universidad. *Comunicar* 33 165–174.

[B6] Azevedo R. (2014). Issues in dealing with sequential and temporal characteristics of self-and socially-regulated learning. *Metacogn. Learn.* 9 217–228. 10.1007/s11409-014-9123-1

[B7] Azevedo R. (2015). Defining and measuring engagement and learning in science: conceptual, theoretical, methodological, and analytical issues. *Educ. Psychol.* 50 84–94. 10.1080/00461520.2015.1004069

[B8] Azevedo R. Behnagh R. Duffy M. Harley J. M. Trevors G. J. (2012). “Metacognition and self-regulated learning in student-centered learning environments,” in *Theoretical Foundations of Student-Center Learning Environments* eds Jonanssen D. Land S. (Mahwah, NJ: Erlbaum) 216–260.

[B9] Azevedo R. Harley J. Trevors G. Duffy M. Feyzi-Behnagh R. Bouchet F. (2013). “Using trace data to examine the complex roles of cognitive, metacognitive, and emotional self-regulatory processes during learning with multi-agent systems,” in *International Handbook of Metacognition and Learning Technologies* eds Azevedo R. Aleven V. (New York, NY: Springer) 427–449.

[B10] Azevedo R. Johnson A. Burkett C. Chauncey A. Lintean M. Cai Z. (2010). “The role of prompting and feedback in facilitating students’ learning about science with MetaTutor,” in *Paper Presented at the 2010 AAAI Fall Symposium Series, Association for the Advancement of Artificial Intelligence* Arlington, VA.

[B11] Azevedo R. Witherspoon A. M. Chauncey A. Burkett C. Fike A. (2009). “MetaTutor: a metacognitive tool for enhancing self-regulated learning,” in *Paper Presented at the AAAI Fall Symposium: Cognitive and Metacognitive Educational Systems* Arlington, VA.

[B12] Balkıs M. (2011). Academic efficacy as a mediator and moderator variable in the relationship between academic procrastination and academic achievement. *Eğit. Araştırmaları Derg.* 45 1–16.

[B13] Bannert M. Reimann P. Sonnenberg C. (2014). Process mining techniques for analysing patterns and strategies in students’ self-regulated learning. *Metacogn. Learn.* 9 161–185. 10.1007/s11409-013-9107-6

[B14] Beswick G. Rothblum E. D. Mann L. (1988). Psychological antecedents of student procrastination. *Aust. Psychol.* 23 207–217. 10.1080/00050068808255605

[B15] Biggs J. B. (2005). *Calidad del Aprendizaje Universitario [Quality of Learning at University].* Madrid: Narcea.

[B16] Bondareva D. Conati C. Feyzi-Behnagh R. Harley J. M. Azevedo R. Bouchet F. (2013). “Inferring learning from gaze data during interaction with an environment to support self-regulated learning,” in *Paper Presented at the International Conference on Artificial Intelligence in Education, AIED 2013* Memphis, TN 10.1007/978-3-642-39112-5_24

[B17] Broadbent J. Poon W. L. (2015). Self-regulated learning strategies & academic achievement in online higher education learning environments: a systematic review. *Internet High. Educ.* 27 1–13. 10.1016/j.iheduc.2015.04.007

[B18] Brusilovsky P. (2001). Adaptive hypermedia. *User Model. User Adapt. Interact.* 11 87–110. 10.1023/A:1011143116306

[B19] Cerezo R. Bernardo A. Esteban M. Sánchez M. Tuero E. (2015). Programas para la promoción de la autorregulación en educación superior: un estudio de la satisfacción diferencial entre metodología presencial y virtual. *Eur. J. Educ. Psychol.* 8 30–36. 10.1016/j.ejeps.2015.10.004

[B20] Cerezo R. Núñez J. C. Rosário P. Valle A. Rodríguez S. Bernardo A. (2010). New media for the promotion of self-regulated learning in higher education. *Psicothema* 22 306–315. 20423637

[B21] Cerezo R. Sánchez-Santillán M. Paule-Ruiz M. P. Núñez J. C. (2016). Students’ LMS interaction patterns and their relationship with achievement: a case study in higher education. *Comput. Educ.* 96 42–54. 10.1016/j.compedu.2016.02.006

[B22] Cherenkova N. Alexandrova N. Chuprina S. (2010). “Methods to assess students’ experiences in an immersive 3D VR environment,” in *Paper Presented at ICL Conference* Hasselt.

[B23] Chmielewski M. R. Grzymala-Busse J. W. (1996). Global discretization of continuous attributes as preprocessing for machine learning. *Int. J. Approx. Reason.* 15 319–331. 10.1016/S0888-613X(96)00074-6

[B24] Choi J. N. Moran S. V. (2009). Why not procrastinate? Development and validation of a new active procrastination scale. *J. Soc. Psychol.* 149 195–212. 10.3200/SOCP.149.2.195-212 19425357

[B25] Chu A. H. C. Choi J. N. (2005). Rethinking procrastination: positive effects of “active” procrastination behavior on attitudes and performance. *J. Soc. Psychol.* 145 245–264. 10.3200/SOCP.145.3.245-264 15959999

[B26] Clariana M. Pros R. C. Martín M. D. M. B. Busquets C. G. (2011). La influencia del género en variables de la personalidad que condicionan el aprendizaje: inteligencia emocional y procrastinación académica. *Rev. Electrón. Interuniv. Formación Profesorado* 14 87–96.

[B27] Cochran J. D. Campbell S. M. Baker H. M. Leeds E. M. (2014). The role of student characteristics in predicting retention in online courses. *Res. High. Educ.* 55 27–48. 10.1007/s11162-013-9305-8

[B28] Cole J. Foster H. (2007). *Using Moodle: Teaching with the Popular Open Source Course Management System.* San Francisco, CA: O’Reilly Media, Inc.

[B29] Corkin D. M. Yu S. L. Lindt S. F. (2011). Comparing active delay and procrastination from a self-regulated learning perspective. *Learn. Individ. Differ.* 21 602–606. 10.1016/j.lindif.2011.07.005

[B30] De Bra P. Calvi L. (1998). AHA! An open adaptive hypermedia architecture. *New Rev. Hypermedia Multimed.* 4 115–139. 10.1080/13614569808914698

[B31] Duffy M. C. Azevedo R. (2015). Motivation matters: interactions between achievement goals and agent scaffolding for self-regulated learning within an intelligent tutoring system. *Comput. Hum. Behav.* 52 338–348. 10.1016/j.chb.2015.05.041

[B32] European Commission (2014). *New Modes of Learning and Teaching in Higher Education.* Luxembourg: European Union.

[B33] Gafni R. Geri N. (2010). Time management: procrastination tendency in individual and collaborative tasks. *Interdiscip. J. Inf. Knowl. Manage.* 5 115–125.

[B34] García E. Romero C. Ventura S. de Castro C. (2009). An architecture for making recommendations to courseware authors using association rule mining and collaborative filtering. *User Model. User-adapt. Interact.* 19 99–132. 10.1007/s11257-008-9047-z

[B35] García E. Romero C. Ventura S. de Castro C. (2011). A collaborative educational association rule mining tool. *Internet High. Educ.* 14 77–88. 10.1016/j.iheduc.2010.07.006

[B36] García-Peñalvo F. J. Seoane Pardo A. M. (2015). Una revisión actualizada del concepto de eLearning. *Educ. Knowl. Soc.* 16 119–144. 10.14201/eks2015161119144

[B37] Goda Y. Yamada M. Kato H. Matsuda T. Saito Y. Miyagawa H. (2015). Procrastination and other learning behavioral types in e-learning and their relationship with learning outcomes. *Learn. Individ. Diff.* 37 72–80. 10.1016/j.lindif.2014.11.001

[B38] Gueorguieva J. M. (2011). *Procrastination a Measurement of Types.* Chicago, IL: University of Illinois at Chicago.

[B39] Häfner A. Stock A. Oberst V. (2015). Decreasing students’ stress through time management training: an intervention study. *Eur. J. Psychol. Educ.* 30 81–94. 10.1007/s10212-014-0229-2

[B40] Hahsler M. Grün B. Hornik K. (2005). A computational environment for mining association rules and frequent item sets. *J. Stat. Softw.* 14 1–25. 10.18637/jss.v014.i15

[B41] Hajizadeh E. Ardakani H. D. Shahrabi J. (2010). Application of data mining techniques in stock markets: a survey. *J. Econ. Int. Finance* 2 109–118.

[B42] Hall M. Frank E. Holmes G. Pfahringer B. Reutemann P. Witten I. H. (2009). The WEKA data mining software: an update. *ACM SIGKDD Explor. Newsl.* 11 10–18. 10.1145/1656274.1656278

[B43] Han J. Kamber M. (2001). *Data Mining Concepts and Techniques.* Waltham, MA: Morgan Kaufmann Publishers.

[B44] Hastie T. Tibshirani R. Friedman J. (2001). *The Elements of Machine Learning: Data Mining, Inference and Prediction.* New York, NY: Springer.

[B45] Hen M. Goroshit M. (2014). Academic procrastination, emotional intelligence, academic self-efficacy, and GPA a comparison between students with and without learning disabilities. *J. Learn. Disabil.* 47 116–124. 10.1177/0022219412439325 22442254

[B46] Hipp J. Güntzer U. Nakhaeizadeh G. (2000). Algorithms for association rule mining - a general survey and comparison. *ACM SIGKDD Explor. Newsl.* 2 58–64. 10.1145/360402.360421

[B47] Hung J. L. Zhang K. (2008). Revealing online learning behaviors and activity patterns and making predictions with data mining techniques in online teaching. *MERLOT J. Online Learn. Teach.* 4 426–437.

[B48] Hussain F. Liu H. Tan C. Dash M. (1999). *Discretization: An Enabling Technique.* Technical Report: TRC6/99. Singapore: School of Computing, National University of Singapore.

[B49] Jacobson C. M. (2008). “Knowledge sharing between individuals,” in *Knowledge Management: Concepts, Methodologies, Tools, and Applications* ed. Jennex M. E. (Philadelphia, PA: IGI Global) 1633–1642. 10.4018/978-1-59904-933-5.ch135

[B50] Karatas H. (2015). Correlation among academic procrastination, personality traits, and academic achievement. *Anthropologist* 20 243–255.

[B51] Kardan A. A. Saryazdi N. G. Mirashk H. (2012). Learner clustering and association rule mining for content recommendation in self-regulated learning. *Int. J. Comput. Sci. Res. Appl.* 2 69–78.

[B52] Katz I. Eilot K. Nevo N. (2014). “I’ll do it later”: type of motivation, self-efficacy and homework procrastination. *Motiv. Emot.* 38 111–119. 10.1007/s11031-013-9366-1

[B53] Kim E. Seo E. H. (2013). The relationship of flow and self-regulated learning to active procrastination. *Soc. Behav. Pers.* 41 1099–1113. 10.2224/sbp.2013.41.7.1099

[B54] Kim J. H. Park Y. Song J. Jo I. H. (2014). “Predicting students’ learning achievement by using online learning patterns in blended learning environments: comparison of two cases on linear and non-linear model,” in *Proceedings of the 7th International Conference on Educational Data Mining* London 407–408.

[B55] Kim K. R. Seo E. H. (2015). The relationship between procrastination and academic performance: a meta-analysis. *Pers. Individ. Dif.* 82 26–33. 10.1016/j.paid.2015.02.038

[B56] Kirk D. Oettingen G. Gollwitzer P. M. (2013). Promoting integrative bargaining: mental contrasting with implementation intentions. *Int. J. Conflict Manage.* 24 148–165. 10.1108/10444061311316771

[B57] Klingsieck K. B. Fries S. Horz C. Hofer M. (2012). Procrastination in a distance university setting. *Distance Educ.* 33 295–310. 10.1080/01587919.2012.723165

[B58] Kotsiantis S. Kanellopoulos D. (2006). Association rules mining: a recent overview. *GESTS Int. Trans. Comput. Sci. Eng.* 32 71–82.

[B59] Lajoie S. P. Azevedo R. (2006). Teaching and learning in technology-rich environments. *Handb. Educ. Psychol.* 2 803–821.

[B60] Lee E. (2005). The relationship of motivation and flow experience to academic procrastination in university students. *J. Genet. Psychol.* 166 5–15. 10.3200/GNTP.166.1.5-15 15782675

[B61] Lee Y. Choi J. Kim T. (2013). Discriminating factors between completers of and dropouts from online learning courses. *Br. J. Educ. Technol.* 44 328–337. 10.1111/j.1467-8535.2012.01306.x

[B62] Lehmann T. Hähnlein I. Ifenthaler D. (2014). Cognitive, metacognitive and motivational perspectives on preflection in self-regulated online learning. *Comput. Hum. Behav.* 32 313–323. 10.1016/j.chb.2013.07.051

[B63] Levy Y. Ramim M. M. (2012). A study of online exams procrastination using data analytics techniques. *Interdiscip. J. E Learn. Learn. Objects* 8 97–113.

[B64] Lewis S. Whiteside A. Dikkers A. G. (2014). Autonomy and responsibility: Online learning as a solution for at-risk high school students. *J. Distance Educ.* 29 1–11.

[B65] Liu H. Hussain F. Tan C. L. Dash M. (2002). Discretization: an enabling technique. *Data Min. Knowl. Discov.* 6 393–423. 10.1023/A:1016304305535

[B66] Liu O. L. Rijmen F. MacCann C. Roberts R. (2009). The assessment of time management in middle-school students. *Personal. Individ. Differ.* 47 174–179. 10.1016/j.paid.2009.02.018

[B67] Lust G. Collazo N. A. J. Elen J. Clarebout G. (2012). Content management systems: enriched learning opportunities for all? *Comput. Hum. Behav.* 28 795–808.

[B68] Lust G. Elen J. Clarebout G. (2013). “Measuring students’ strategy-use within a CMS supported course through students’ tool-use patterns,” in *Proceedings of the Book of Abstracts 15th Biennial Conference EARLI 2013* Munich 571–572.

[B69] Macfadyen L. P. Dawson S. (2012). Numbers are not enough. Why e-learning analytics failed to inform an institutional strategic plan. *Educ. Technol. Soc.* 15 149–163.

[B70] Michinov N. Brunot S. Le Bohec O. Juhel J. Delaval M. (2011). Procrastination, participation, and performance in online learning environments. *Comput. Educ.* 56 243–252. 10.1016/j.compedu.2010.07.025

[B71] Mora N. Caballé S. Daradoumis T. Barolli L. Kulla E. Spaho E. (2014). “Characterizing social network e-assessment in collaborative complex learning resources,” in *Proceedings of the Complex, Intelligent and Software Intensive Systems (CISIS), 2014 Eighth International Conference on IEEE* Washington, DC 257–264. 10.1109/CISIS.2014.36

[B72] Mosharraf M. Taghiyareh F. (2012). “Improving student success rates through a semi-personalized feedback system,” in *Proceedings of the 11th European Conference on e-Learning: ECEL* Prague 364–369.

[B73] Murray M. C. Pérez J. Geist D. B. Hedrick A. (2012). Student interaction with online course content: build it and they might come. *J. Inf. Technol. Educ.* 11 125–140.

[B74] Murugananthan V. ShivaKumar B. L. (2016). An adaptive educational data mining technique for mining educational data models in elearning systems. *Indian J. Sci. Technol.* 9 1–5. 10.17485/ijst/2016/v9i3/86392

[B75] Nahar J. Imam T. Tickle K. S. Chen Y. P. P. (2013). Association rule mining to detect factors which contribute to heart disease in males and females. *Expert Syst. Appl.* 40 1086–1093. 10.1016/j.eswa.2012.08.028

[B76] Núñez J. C. Cerezo R. Bernardo A. Rosário P. Valle A. Fernández E. (2011). Implementation of training programs in self-regulated learning strategies in Moodle format: results of a experience in higher education. *Psicothema* 23 274–281. 21504681

[B77] Odacı H. Kalkan M. (2010). Problematic Internet use, loneliness and dating anxiety among young adult university students. *Comput. Educ.* 55 1091–1097. 10.1016/j.compedu.2010.05.006

[B78] Paredes W. C. Chung K. S. K. (2012). “Modelling learning & performance: a social networks perspective,” in *Proceedings of the 2nd International Conference on Learning Analytics and Knowledge* Vancouver, BC 34–42. 10.1145/2330601.2330617

[B79] Patrzek J. Sattler S. Van Veen F. Grunschel C. Fries S. (2015). Investigating the effect of academic procrastination on the frequency and variety of academic misconduct: a panel study. *Stud. High. Educ.* 40 1014–1029. 10.1080/03075079.2013.854765

[B80] Paule-Ruiz M. P. Riestra-Gonzalez M. Sánchez-Santillan M. Pérez-Pérez J. R. (2015). The Procrastination related indicators in e-learning platforms. *J. Univ. Comput. Sci.* 21 7–22.

[B81] Rabin L. A. Fogel J. Nutter-Upham K. E. (2011). Academic procrastination in college students: the role of self-reported executive function. *J. Clin. Exp. Neuropsychol.* 33 344–357. 10.1080/13803395.2010.518597 21113838

[B82] Rakes G. C. Dunn K. E. (2010). The impact of online graduate students’ motivation and self-regulation on academic procrastination. *J. Interact. Online Learn.* 9 78–93.

[B83] Reid M. J. Moore J. L. (2008). College readiness and academic preparation for postsecondary education oral histories of first-generation urban college students. *Urban Educ.* 43 240–261. 10.1177/0042085907312346

[B84] Rice W. H. (2006). *Moodle E-Learning Course Development A Complete Guide to Successful Learning Using Moodle.* Birmingham: Packt Publishing.

[B85] Rodríguez-Málaga L. Cerezo R. Rodriguez C. (2017). “Metacognition and learning disabilities in higher education,” in *Learning Disabilities: Assessment, Management and challenges* ed. Pearson R. (New York, NY: Nova Science) 25–60.

[B86] Romero C. Espejo P. G. Zafra A. Romero J. R. Ventura S. (2013). Web usage mining for predicting final marks of students that use Moodle courses. *Comput. Appl. Eng. Educ.* 21 135–146. 10.1002/cae.20456

[B87] Romero C. Romero J. R. Luna J. M. Ventura S. (2010a). “Mining rare association rules from e-learning data,” in *Proceedings of the 3rd International Conference on Educational Data Mining* Pittsburgh, PA 171–181.

[B88] Romero C. Ventura S. Vasilyeva E. Pechenizkiy M. (2010b). “Class association rules mining from students’ test data,” in *Proceedings of the 3rd International Conference on Educational Data Mining* Tarrytown, NY 317–319.

[B89] Romero C. Ventura S. (2013). Data mining in education. *Wiley Interdiscip. Rev.* 3 12–27. 10.1002/widm.1075

[B90] Romero C. Ventura S. García E. (2008). Data mining in course management systems: moodle case study and tutorial. *Comput. Educ.* 51 368–384. 10.1016/j.compedu.2007.05.016

[B91] Romero M. (2013). “Comparing procrastination in arts, sciences, technology, social sciences, and humanities high school students,” in *Proceedings of the EDULEARN13: 5th International Conference on Education and New Learning Technologies* Barcelona 3465–3465.

[B92] Sánchez A. M. (2010). Procrastinación académica: un problema en la vida universitaria. *Studiositas* 5 87–94.

[B93] Sánchez-Santillán M. Paule-Ruiz M. Cerezo R. Alvarez-García V. (2016). MeL: modelo de adaptación dinámica del proceso de aprendizaje en eLearning. *Anal. Psicol.* 32 106–114. 10.6018/analesps.32.1.195071

[B94] Scheffer T. (2001). “Finding association rules that trade support optimally against confidence,” in *Proceedings of the European Conference on Principles of Data Mining and Knowledge Discovery* (Berlin: Springer) 424–435. 10.1007/3-540-44794-6_35

[B95] Schwarz N. (1999). Self-reports: how the questions shape the answers. *Am. Psychol.* 54 93–105. 10.1037/0003-066X.54.2.93

[B96] Sekhavatian A. Mahdavi M. (2011). “Application of recommender systems on e-learning environments,” in *Proceedings of the EDULEARN11: 3rd International Conference on Education and New Learning Technologies* Barcelona 2679–2687.

[B97] Shukla N. J. Hassani H. Casleton R. (2014). “A comparison of delivery methods for distance learning mathematics courses,” in *Paper Presented at SoTL Commons Conference* Savannah, GA.

[B98] Steel P. (2007). The nature of procrastination: a meta-analytic and theoretical review of quintessential self-regulatory failure. *Psychol. Bull.* 133 65–94. 10.1037/0033-2909.133.1.65 17201571

[B99] Steel P. Klingsieck K. B. (2016). Academic procrastination: psychological antecedents revisited. *Aust. Psychol.* 51 36–46. 10.1111/ap.12173

[B100] Stoeger H. Ziegler A. (2008). Evaluation of a classroom based training to improve self-regulation in time management tasks during homework activities with fourth graders. *Metacogn. Learn.* 3 207–230. 10.1007/s11409-008-9027-z

[B101] Sureda-Negre J. Comas-Forgas R. Oliver-Trobat M. F. (2015). Academic plagiarism among Secondary and High School Students: differences in gender and procrastination/plagio académico entre alumnado de secundaria y bachillerato: diferencias en cuanto al género y la procrastinación. *Comunicar (English edition)* 22 103–110. 10.3916/C44-2015-11

[B102] Talavera L. Gaudioso E. (2004). “Mining student data to characterize similar behavior groups in unstructured collaboration spaces,” in *Proceedings of the European Conference on Artificial Intelligence, Workshop: Artificial Intelligence in Computer Supported Collaborative Learning (ECAI 2004)* Valencia 17–23.

[B103] Tan C. X. Ang R. P. Klassen R. M. Yeo L. S. Wong I. Y. Huan V. S. (2008). Correlates of academic procrastination and students’ grade goals. *Curr. Psychol.* 27 135–144. 10.1007/s12144-008-9028-8

[B104] Tan P. Steinbach M. Kumar V. Potter C. Klooster S. Torregrosa A. (2001). “Finding spatio-temporal patterns in earth science data,” in *Proceedings of the Knowledge Discovery and Data Mining2001 Workshop on Temporal Data Mining* Vol. 19 New York, NY.

[B105] Terry K. P. S. (2002). *The Effects of Online Time Management Practices on Self-Regulated Learning and Academic Self-Efficacy.* Doctoral dissertation, Virginia Polytechnic Institute and State University Blacksburg, VA.

[B106] Trevors G. Duffy M. Azevedo R. (2014). Note-taking within MetaTutor: interactions between an intelligent tutoring system and prior knowledge on note-taking and learning. *Educ. Technol. Res. Dev.* 62 507–528. 10.1007/s11423-014-9343-8

[B107] Trevors G. Feyzi-Behnagh R. Azevedo R. Bouchet F. (2016). Self-regulated learning processes vary as a function of epistemic beliefs and contexts: mixed method evidence from eye tracking and concurrent and retrospective reports. *Learn. Instr.* 42 31–46. 10.1016/j.learninstruc.2015.11.003

[B108] Tuckman B. W. (2005). Relations of academic procrastination, rationalizations, and performance in a web course with deadlines. *Psychol. Rep.* 96 1015–1021. 10.2466/pr0.96.3c.1015-1021 16173372

[B109] Visser L. B. Korthagen F. A. J. Schoonenboom J. (2015). Influences on and consequences of academic procrastination of first-year student teachers. *Pedagog. Stud.* 92 394–412.

[B110] Wei C. P. Chiu I. T. (2002). Turning telecommunications call details to churn prediction: a data mining approach. *Expert Syst. Appl.* 23 103–112. 10.1016/S0957-4174(02)00030-1

[B111] Winne P. H. Baker R. S. (2013). The potentials of educational data mining for researching metacognition, motivation and self-regulated learning. *JEDM J. Educ. Data Min.* 5 1–8.

[B112] Winters F. I. Greene J. A. Costich C. M. (2008). Self-regulation of learning within computer-based learning environments: a critical analysis. *Educ. Psychol. Rev.* 20 429–444. 10.1007/s10648-008-9080-9

[B113] Yaakub N. F. (2000). *Procrastination among students in institutes of higher learning: Challenges for k-economy. The School of Languages and Scientific Thinking, Universiti Utara Malaysia* Vol. 10 Available at: http://mahdzan.com/papers/procrastinate/default.asp

[B114] Yang J. (2012). *Research on the Present Situation Procrastination Behavior of Primary School Students.* Guizhou: Journal of Xingyi Normal University for Nationalities 2.

[B115] You J. W. (2015). Examining the effect of academic procrastination on achievement using LMS data in e-learning. *Educ. Technol. Soc.* 18 64–74.

[B116] You J. W. (2016). Identifying significant indicators using LMS data to predict course achievement in online learning. *Internet High. Educ.* 29 23–30. 10.1016/j.iheduc.2015.11.003

[B117] Zimmerman B. Risemberg R. (1997). “Self-regulatory dimensions of academic learning and motivation,” in *Handbook of Academic Learning: Construction of Knowledge* ed. Phye G. D. (San Diego, CA: Academic Press) 106–121.

[B118] Zimmerman B. J. (1990). Self-regulated learning and academic achievement: an overview. *Educ. Psychol.* 25 3–17. 10.1207/s15326985ep2501_2

[B119] Zimmerman B. J. (1998). Academic studying and the development of personal skill: a self-regulatory perspective. *Educ. Psychol.* 33 73–86. 10.1080/00461520.1998.9653292

